# Assessment of Evidence-Based Practice (EBP) among physiotherapists in Cameroon: a cross-sectional survey

**DOI:** 10.1186/s12909-024-05273-w

**Published:** 2024-03-22

**Authors:** Dilane Landry Nsangou Muntessu, Hyacinte Trésor Ghassi, Franklin Chu Buh, Ange Wandji Nietho, Julio Rostan Siewe, Mpatoutou Me Mpatoutou

**Affiliations:** 1 Physiotherapy Unit, Protestant Hospital of Bafoussam, Bafoussam, Cameroon; 2 Department of Physiotherapy, University and Strategic Institute of the Estuary, (IUEs/Insam), Bafoussam, Cameroon; 3 Department of Physiotherapy, Faculty of Medicine and Pharmaceutical Sciences, University of Dschang, Dschang, Cameroon; 4 Department Animal Biology, University of BUEA, Buea, Cameroon; 5 FOYAGUEM Higher Institute, Dschang, Cameroon; 6 Hope Medical Clinic, Bertoua, Cameroon

**Keywords:** Evidence-based practice, Physiotherapists, Assessment, Domains, Cameroon

## Abstract

**Background:**

Evidence-Based Practice (EBP) is reported to ease effective and adequate decision making for all works of life including health professionals. Investigating the level of implementation of EBP among physiotherapists helps to identify barriers and propose solutions for its extensive implementation. Despite available data on EBP elsewhere, it remains limited or non-existent in Cameroon. This study was designed to establish the current state of EBP among physiotherapists in Cameroon, by assessing knowledge, practice, and barriers to its implementation.

**Methods:**

A cross-sectional survey was conducted online among French- and English-speaking physiotherapists in Cameroon over a period of six months from April to July 2023, using the EBP^2^ questionnaire. This enabled us to collect socio-demographic data from participants and information on their knowledge, understanding and practice of EBP and possible barriers to EBP according to 5 domains (Confidence, Relevance, Terminology, Practice, Compatibility) scored out of 100. Data were analysed using IBM SPSS 25.0 software and Pearson correlations (95% CI) and significance (*p* < 0.05).

**Results:**

A total of 110 physiotherapists practising in the 10 regions of Cameroon participated in the study. The majority were male (54.5%), the median age was 34 years (age range 20 to 50), the median year of graduation was 2019 (range 2004 to max 2022) with 72.7% never having knowledge or training in EBP. Participants scored below 50/100 for 3 domains (confidence, relevance, and compatibility) showing poor general knowledge and understanding of EBP, although they generally had a positive attitude towards EBP. The use of EBP in practice was low (26.3/100 SD. 6.5), despite that they appeared to have a good understanding of research terminology (55.4 SD. 17.2). Level of study of participants did not appear to significantly influence domain scores (*P* > 0.05). The main barriers to practice were lack of time (75.1%), workload (66%), cost of access to information resources like databases for seek informations about recent support protocols (60%), ease of access to computers (49%), and lack of resources or skills (49%).

**Conclusion:**

Physiotherapists in Cameroon have a poor knowledge of EBP and a low level of practice of EBP, despite an overall positive attitude towards it. These results could inform stakeholders of higher education on the need to improve training of physiotherapy students in the domain of EBP in Physiotherapy. Also, it will help in raising the level of scientific research and promoting the implementation of EBP in Cameroon.

## Introduction

The concept of “Evidence-based Medicine” (EBM) was introduced into scientific literature in 1991 by Professor David Laurence Sackett and colleagues and is defined as “the conscientious and judicious use of the best current clinical research evidence in the personalised management of patients” [1]. On one hand, promoters of the concept question the foundations of practical knowledge based on intuition, the use of unsystematic observations derived from clinical experience, and the pathophysiology and mechanism of diseases. On the other hand, they reject the authority of former masters and experts, who were responsible for recommendations deemed to be invalid [1, 2]. In the space of a few years, this concept has been used throughout the world, and is now structured into 5 stages: formulation of a resolvable clinical question, systematic search for the best available evidence in the scientific literature, critical reading of the data obtained from the scientific literature, application of the results obtained from the previous two stages to the clinical situation encountered, and evaluation of efficacy [3].

As in medicine, all other health professions, such as physiotherapy, have been encouraged to use the principles of EBM, which has been renamed EBP (Evidence-Based Practice), to guide their therapeutic decisions [4]. Related to the practice of physiotherapy, several EBP guidelines have been published over the last twenty years. However, initial studies into the implementation of EBP by physiotherapists have shown that they spend very little time reading scientific articles in the field and use techniques learnt during their initial training or based on experience gained during their practice to manage patients [5].

Several self-administered questionnaires were developed to assess the knowledge, perceptions, skills, use and barriers to EBP among physiotherapists, and it emerged that lack of training in EBP, lack of time and skills including the use of databases such as PEDro, pubmed, medline, google scholar, scopus were identified as the main barriers to EBP among physiotherapists. This is despite their positive attitudes and interest in the concept of EBP in general [6, 7]. Other systematic reviews found that the main barriers to the use of EBP by physiotherapists were lack of time, accessibility of data, language, and shortcomings in research methodology [8–10].

Adequate education of physiotherapists on EBP may limit some of these barriers and promote its implementation. Scurlock-Evans et al. [9] found a positive correlation between education, knowledge, positive attitudes and barriers to EBP. Other recent questionnaire surveys support these reviews, noting that physiotherapists think EBP is very important and useful for clinical practice, despite several barriers to its implementation in France [11], Italy [12], Saudi Arabia [13], Philippines [14], United Arab Emirates [15], Canada [16] and Australia [17].

Despite several studies elsewhere, data on EBP in Sub-Saharan Africa and Cameroon in particular are limited or non-existent. Furthermore, only few studies have reported a detailed inventory of EBP implementation among physiotherapists including the knowledge,practice and barriers that prevent its implementation. An attempt in this direction was carried out among health science students by Ngeh (2019) in Cameroon and Kishna (2022) in Central Africa [18, 19] in which it was noted that the inadequacy and poverty of research infrastructures, research which is difficult probably because of the lack of funding and supervision, and a great shortage of local scientific journals contribute to the low level of these students in research. Nevertheless, to the best of our knowledge, no publication to date has focused specifically on the participation of physiotherapists in EBP in Cameroon [19]. This is the first study on this topic.

The relevance of this study in Cameroon will be to determine an overview of the knowledge, attitudes, perceptions, education and practice of physiotherapists in EBP. The reason being it is not only a principal element to improve the performance of physiotherapists and the physiotherapy profession on a national level, but also for the training of future physiotherapists in Cameroon and the sub-region. This will bring Cameroon physiotherapists up to date with EBP as those in Europe, America and other developed countries [11]. The aim of this cross-sectional study was therefore to establish the current state of evidence-based practice (EBP) among physiotherapists in Cameroon, by assessing their knowledge, perception and potential barriers for the use of EBP.

## Methods

### Description of study, area and study population

This was a cross-sectional study on physiotherapists practising in Cameroon. Cameroon is a Central African country located at the bottom of the Gulf of Guinea with an estimated population of 28.647.293 in 2023 spread over 10 regions [20, 21]. To practise as a physiotherapist in Cameroon, you need to complete a training course for 3 to 4 years (initial training of 6 to 8 semesters). This will enable you to obtain a diploma, which can be the “Higher National Diploma” (HND) for anglophones, a “Brevet de Technicien Supérieur en Santé (BTS)” for francophones, or the senior medical and health technician diploma, physiotherapy option in three years and a bachelor's degree in the fourth year in a training school approved by or under the supervision of the Ministry of Public Health (MINSANTE) or the Ministry of Higher Education (MINESUP) [22].

This study included French-speaking and English-speaking physiotherapists of both sexes practising in the 10 regions of Cameroon what are The North, the Far-North, Adamawa, the South, the East, the Center, the West, the Coast, the Northwest and the Southwest who had at least 1 year’s work experience. Were excluded, physiotherapists who were not available at the time of data collection and questionnaires returned with incomplete information.

### Sampling

The target population consisted of all physiotherapists practising in the 10 regions of Cameroon. We used a convenience sampling technique, as participants were recruited according to their availability and accessibility. They were recruited online, through social networks or by email and telephone calls. The survey period ran from April to July 2023.We contacted all the regional representatives of Cameroon Society of Physiotherapy (CASP) and sent them the questionnaire in the form of a “google forms” link,and we also contacted the physiotherapists individually who in turn transmitted the questionnaire to other colleagues.

Here is the link to the French version of the questionnaire

https://docs.google.com/forms/d/e/1FAIpQLSfSU-e582iJD7yJQD_BT7ywD2e5SjoXs2C9e0v1Dd342RlRrg/viewform?usp=sf_link

And for the English version

https://docs.google.com/forms/d/e/1FAIpQLSeJBo1iZTLBsGOnyOAxK88ZPAkDg91ot6NcuDgfV-ozdacVMA/viewform?usp=sf_link

### Data collection tool

We used the French translation of McEvoy's EBP2 questionnaire, which was first used in a study of the EBP profiles of physiotherapists in France [11], and made it available online, and the original English version for English-speaking physiotherapists. The questionnaire consisted of 86 questions, 74 of which were specific to the evaluation of EBP based on 6 domains (Confidence, Competence, Practice, Relevance, Terminology and Other Characteristics).

The socio-demographic data covered 12 questions and included age, gender, year of graduation, country of practice, region of practice, highest degree obtained, country of initial training, occupation, work pattern, student or non-student status, specialisation and previous participation in EBP training.

These areas explore:

- The importance that participants attach to the EBP (relevance section, 14 questions);
- Understanding of terms commonly used in research (terminology section, 17 questions);
- Each participant’s perception of his or her ability to use EBP (confidence section, 11 questions);
- The use of EBP in clinical situations (practice domain, 9 questions);
- Perception of the compatibility of EBP with the professional environment (compatibility domain, 7 questions).

The remaining 16 questions were in the “other characteristics” group. Scores ranging from 0 to 100 were assigned to each domain, and a score below 50 is considered poor in relation to the domain concerned. A score of 50 and above indicates a positive score in the area concerned.

The EBP2 questionnaire was tested and found to be reliable as an Evidence-based practice assessment tool, with the following metric values obtained; Correlation coefficient *r* = 0.54- 0.80, Statistical significance (P less than 0.05), Cronbach’s alpha = 0.96 [11].

To ensure that the terms were understood, the questionnaire was pre-tested with approximately 10 final year physiotherapy students, who were not included in the study.

### Data analysis

The data were analysed using IBM SPSS Version 25.0 software and the statistical significance was set at *P* < 0.05, 95% CI. Descriptive statistics characterised the socio-demographic data of physiotherapists and their distribution over the national territory and to analyze proportions in percentages related to the knowledge of physiotherapists on EBP and the challenges to the practice of EBP. Pearson correlation coefficient was calculated to look for a linear correlation between the domains (relevance, compatibility, practice, confidence, terminology) and certain socio-demographic data as level of study and year of graduation.

## Results

### Sociodemographic data

During the four months of the survey, 110 physiotherapists from the 10 regions of the country participated in the study.

Most of the respondents were male (54.5%) with an average age of 34 years, the median year of graduation in physiotherapy was 2019 and 95.5% had been trained in Cameroon. Most participants practised in the French-speaking regions of the country, mainly in the Littoral, West and Centre (26.3%, 26.3% and 18.1%, respectively). The least represented regions were the South, North-West, South-West and Far North: 1.8%, 3.6%, 3.6% and 3.6% respectively. Most (41.8%) of the physiotherapists held a Bachelor’s degree. About 45% of participants had no specialised certification. For physiotherapists with certification, 24.4% had sports medicine certification and 23.6% manual therapy certification. Majority (72.7%) of physiotherapists had not received EBP training (Table 1).

**Table 1:** Socio-demographic data

Data	Characteristics
Age (years)	Med. = 34
	Min = 20
	Max = 50
**Year of graduation**	Med. = 2019
	Min = 2004
	Max = 2022
**Sex**	Male = 54.5%
	Female = 45.5% (SR = 1.2)
**Country of formation**	Cameroon = 95.5%
	Others = 4.5% (Ghana, Morocco,South Africa)
**Region of work:**	
Adamawa	5 (4.5%)
Nord	5 (4.5%)
Far-Nord	4 (3.6%)
Littoral	29 (26.3%)
West	29 (26.3%)
Nord-West	4 (3.6%)
South-West	4 (3.6%)
East	8 (7.2%)
Centre	20 (18.1%)
South	2 (1.8%)
Total	110
**Main working environment**	
Liberal	39 (35.5%)
Sportive Clubs	15 (13.6%)
Sportive federation	5 (4.5%)
Public Hospital	24 (21.8%)
Private clinic	46 (41.8%)
Academy	13 (11.8)
Para public hospital	1 (0.9%)
**Certification post- initial formation**	
Sports physiotherapy (DU or equivalent)	27 (24.5%)
Paediatric	5 (4.5%)
Orthopaedic	10 (9.1%)
Musculoskeletal	16 (14.5%)
Cardiovascular/respiratory	2 (1.8%)
Education’s sciences	4 (3.6%)
Neurology	3 (2.7%)
Manual therapy	26 (23.6%)
Gynecology	1 (0.9%)
None	49 (44.5%)
**Are you following a continuous program?**	
Yes	43 (39%)
No	67 (60.9%)
**Highest diploma**	
Doctorate	1 (0.9%)
Masters	27 (24.5%)
bachelor	46 (41.8%)
HND	30 (27.3%)
Bachelor fellow	6 (5.5%)
Total	110 (100%)
**Formation to EBP**	
In the initial program (> 20 h)	2 (1.8%)
As a continuing program (> 20 h)	5 (4.5%)
Very short courses (10-20 h)	3 (2.7%)
Courses during week-ends (3-10 h)	6 (5.5%)
A conference or seminar (1-3 h)	14 (12.7%)
Never	80 (72.7%)

### Knowledge of physiotherapists on EBP

#### General data

Forty-seven physiotherapists (42.7%) did not understand what is meant by “evidence-based practice”, 58 physiotherapists (53%) were not aware of recent EBP developments in the profession. Twenty-nine physiotherapists (26%) definitely intend to improve their knowledge of EBP, 25% might consider it and 33% very likely to consider it; 79 physiotherapists (72%) intend to apply the results of the best available evidence to improve their practice.

#### EBP domain scores

Physiotherapists as a whole have a fairly good knowledge of the terminologies commonly used in research (score of …), despite the fact that they seem to have little confidence in their ability to use EBP (score of 36.52/100). In addition, they hardly use EBP in their clinical practice (score of 26.31/100) and don’t attach much importance to it (relevance score = 33.23/100). They also don’t have a good perception of EBP's compatibility with the professional environment (compatibility score = 24.06/100) (Table 2).

**Table 2:** Scores by EBP domains for the Cameroonian physiotherapists

Domains	Scores ± Standard Deviation
Confidence	36.5 (± 13.1)
Practice	26.3 (± 6.5)
Pertinence	33.2 (± 11.3)
Compatibility	24.06 (± 8.08)
Terminology	55.4 (± 17.2)
Other characteristics	41.03 (± 8.85)

#### Pearson correlation coefficient between the different domains

There is a positive and statistically significant correlation between physiotherapist’s confidence in their practice of EBP (*r* = 0.33, *P* = 0.00), confidence and terminology (*r* = 0.23, *P* = 0.012), compatibility and relevance (*r* = 0.34, *P* = 0. 00), meaning that the more confident physiotherapists were in their EBP skills, the more they practiced EBP and the more compatible they found EBP in their professional environment, the better their understanding of research terminology and the more they practiced EBP. Furthermore, there was a negative correlation between EBP practice and relevance (*r* = -0.30, *P* = 0.001); relevance and terminology (*r* = -0.25, *P* = 0.007); this means that the more relevant they found EBP to be, the less they practiced, and the more familiar they were with the terms used in research, the less relevant they found EBP to be (Table 3).

**Table 3:** Correlation between different domains of implementation of EPB among physiotherapists

EBP Domains	Confidence	Practice	Pertinence	Compatibility	Terminology
Confidence	-				
Practice	*r* = 0.33 (*P* = 0.00*)	-			
Pertinence	*r* = -0.164 (*P* = 0.086)	*r* = -0.30 (*P* = 0.001)	-		
Compatibility	*r* = 0.024 (*P* = 0.08)	*r* = -0.11 (*P* = 0.2)	*r* = 0.34 (*P* = 0.00*)	-	
Terminology	*r* = 0.23 (*p* = 0.012*)	*r* = 0.16 (*P* = 0.08)	*r* = -0.25 (*P* = 0.007*)	*r* = 0.12 (*P* = 0.1)	-

"*" means that the result is statistically significant

#### Correlation between EBP domains, level of study and year of graduation

As shown in Table 4, there is a weak negative correlation between the level of education and the importance that physiotherapists accord to the EBP (relevance domain) meaning that the higher the level of education, the less physiotherapists find the EBP relevant (*r* = -0.18 *P* = 0.04). Furthermore, the more recent the initial training diploma, the less physiotherapists perceive the EBP to be compatible with the professional environment (*r* = -0.37, *P* = 0.0001*). Similarly, the more recent the diploma, the less physiotherapists know the terms generally used in research (*r* = -0.2, *P* = 0.012*).

**Table 4:** Correlation between different EBP domains and the level of study

EBP domains	Correlation with the level of study (r)	*P* value	Correlation with the years of the diploma (r)	*P* value
Terminology	0.088	**0.36**	-0.2	0.012*
Confidence	0.1	0.29	0	0.94
Practice	0.15	0.1	-0.002	0.98
Compatibility	0.06	0.4	-0.37	0.0001*
Pertinence	**-0.18**	**0.04**	0.11	0.23

"*" means that the result is statistically significant

#### Correlation between knowledge of EBP and area of practice among participants

Findings from this study indicate that the understanding of EBP, the knowledge of recent developments of EBP in physiotherapy and the domain of relevance vary in the same direction (*r* = 0.36, *p* = 0.0001, *r* = 0. 34, *p* = 0.0001, respectively); whereas they vary in the opposite direction regarding the practical domain; *r* = -0.35; *p* = 0.000, *r* = -0.28, *p* = 0.002, respectively). This mean that despite the good understanding that some physiotherapists have of EBP and the fact that some of them are aware of current developments in EBP in physiotherapy, they still do not use EBP in practice (Table 5).

**Table 5:** Correlation between knowledge and domain of practice and relevance of EBP

	Domain relevance	*P* value	Domain practice	*P*-value
Participant understands EBP	*r* = 0.36	0.0001*	*r* = -0.35	0.000*
Participant aware of actual development of EBP in my profession	*r* = 0.34	0.0001*	*r* = -0.28	0.002*

"*" means that the result is statistically significant

### Barriers and motivations to the use of EBP by physiotherapists in Cameroon

Table 6 presents the main barriers to the implementation of EBP that were identified. These are: lack of time (75.1% of physiotherapists agree), workload (66% of physiotherapists agree), cost of access to information resources (60% of physiotherapists agree), ease of access to computers (49% of physiotherapists agree), and lack of resources (49% of physiotherapists agree). Collective support from colleagues and support from managers were the main motivators for using EBP.

**Table 6:** Barriers and motivations to EBP among Cameroonian physiotherapists

	Totally disagree	Disagree	Neutral	Agree	Totally agree
Lack of time	7	5	15	37 (33.6%)	46 (41.8%)
The workload	7	13	17	36 (32.7%)	37 (33.6%)
Information resources coast	10	10	24	32 (29%)	34 (30.9%)
Easy access to a computer	9	22	25	31 (28.1%)	23 (20.9%)
Participants have enough resources	33 (30%)	21 (19%)	19	25	12
**Motivations to the use of EBP**					
Collective support from colleagues	28	9	25	30 (27.2%)	18 (16.3%)
Executive support	30	11	21	29 (26.3%)	19 (17.2%)

## Discussion

To the best of our knowledge, this study is the first to present an overview of the practice of EBP by physiotherapists in Cameroon. A total of 110 physiotherapists from all 10 regions of the country participated in the study. Most respondents were male (54.5%), the median age was 34 years, the median year of graduation was 2019 and the majority (41.8%) practised in private clinics. The gender distribution in our sample seems to contradict the national distribution for 2022 published by WP (World Physiotherapy), in which 80% of physiotherapists in Cameroon were female [22] , perhaps because they were more available during this period than women during the study’s period. In addition, 44.5% of participants had no certified specialisation and 80% said they had never been trained in EBP. These data appear to be contrary to the literature [11], which is not a surprise because training in various domains of specialisation in physiotherapy (such as sport, paediatrics, manual therapy, gynaecology, neurology…) are rare and expensive in Cameroon, but also the initial training programs for physiotherapists most often does not include EBP.

Concerning the knowledge of physiotherapists about EBP, the results of the study show that 43% of physiotherapists do not understand what is meant by EBP, 53% are not aware of recent developments of EBP in the profession which reflects a lack of knowledge about EBP. This result does not corroborate with the study by Al Ketbi et al. [15] in the United Arab Emirates (UAE), who found that the majority (84.9%) of physiotherapists who took part in the study had a good knowledge of EBP. This difference could be explained by the fact that the majority of physiotherapy training schools in Cameroon do not include EBP courses in their training curricula. The difference could also be due to variation in the data collection tool used in the two studies. Also, contrary to our study, the study by Aweto et al. [23] in Nigeria showed that physiotherapists in 14 Nigerian states had a good knowledge of EBP. This difference could be explained by the fact that the profession in general is less established and established in Cameroon than in Nigeria.

Considering the scores by domain of the EBP2 questionnaire, we deduce that physiotherapists do not attach great importance to the EBP and insufficiently implement it in practice. On the other hand, they seem to have a good knowledge of the terminology of EBP, and the scores for the other domains varies between 24 and 41 (relevance, compatibility and other characteristics), which suggests a need for improvement. This is in line with the study by Hasani et al. [13] in Saudi Arabia where they reported evidence-based practiced was not extensively implemented among physiotherapists in Saudi Arabia. However, our results on the importance accorded to EBP is not in line with that of Hasani et al. [13] as they reported that up to half of the physiotherapists in their study accorded importance of EBP in clinical practice, contrary to our study where most of them did not give enough importance to implementing EBP. This difference can be explained by the differences in sample sizes, as we worked with 110 physiotherapists against only 64 in their study. Furthermore, there is a positive correlation between compatibility and relevance and between terminology and confidence. We also note specifically that confidence in their abilities is linked to practice (*r* = 0.33), as also shown in the study conducted by AlKetbi et al. [15] in the UAE. Physiotherapists, although finding EBP relevant, don’t practise it as much (negative correlation between the practice of EBP and its relevance); this can perhaps be explained by laziness or lack of time to search for evidence in the literature and apply it in daily care. Also, a negative correlation was found between relevance and terminology, showing that knowledge of the terms used in research is not favourable to the fact that participants find EBP relevant. This can be explained by the general lack of knowledge on the fundamentals of EBP that was observed. Furthermore, the level of education does not seem to favour the participants' understanding of the relevance of EBP, probably due to the fact that there are no modules on EBP in the training curricula. physiotherapy students.

In contrast, a study of the EBP profile of physiotherapists in France found a positive correlation between the 5 domains (practice, terminology, confidence, relevance and compatibility) of EBP2 taken in pairs [11]. This difference could be due to the fact that physiotherapists in France have been trained in EBP in their initial training since 2015 [11], and also the profession is more advanced, which is not the case in Cameroon given that the majority of schools do not have specific EBP courses and seminars on EBP among practicing physiotherapists are rare. However, our study provides a detailed overview of the current situation of EBP among physiotherapists in Cameroon which will permit us to draw adequate conclusions which will help in developing strategies in training on and implementing EBP among physiotherapists in Cameroon and in the sub-region.

As barriers to EBP, we noted lack of time; workload; cost of access to information resources; ease of access to computers and lack of resources. This could be explained by the fact that most physiotherapists in Cameroon are not trained on EBP. Therefore, they do not have adequate skills on the use of scientific databases such as PEDro, pubmed, medline, google scholar, scopus, to find evidence in daily practice. This encourages a certain laxity in finding sufficient evidence for designing and choosing the most effective treatment modalities. Similar barriers have been observed in studies of physiotherapists in different countries such as France and the United Arab Emirates, as also shown in the literature [11, 15]. However, findings from this study slightly differ with that of a systematic review by Paci et al. [10] who found that organisational issues and methodological skills were the main barriers to the implementation of EBP among physiotherapists. This difference could be explained by differences in study design and methodology; for example, in some articles used in this literature review, the authors did not use the EBP2 questionnaire as we did here; furthermore, Sub-analyses were performed grouping studies based on countries where surveys were performed, classified as either developed or developing countries. In addition, we found that collective support from colleagues and managers were the main motivating factors for the use of EBP. This could perhaps be explained by the fact that it is obvious that if physiotherapists join forces to help each other by informing each other of recent advances in the field for example during scientific debates in seminars this will certainly be source of encouragement for EBP; Also, managers who are employers have the possibility of directly influencing the professional activity of the physiotherapists under their authority, for example through financial rewards and various promotions.

### Strengths and limitations of the study

A major strength of this study is that it addresses for the first time in Cameroon the use of EBP among physiotherapists. Also, the study outlines the correlations between knowledge of EBP, and its relevance and practice, as well as outlines the barriers to implementing EBP. This will help take effective and appropriate measures to ensure the implementation of EBP among physiotherapists in Cameroon. Among the limitations of our study, we highlight the low participation of several regions such as the North, the Far North, the North West, the South West and the South. Furthermore, we cannot say with certainty that our results show an effective inventory of the EBP of all physiotherapists in Cameroon, because of the relatively high risk of bias due to online surveys added to the fact that we don’t have the assurance that the participation percentages of physiotherapists of both sexes are representative of reality, due to the absence of a reliable directory of the exact distribution of practitioners in the country. Apart from these limitations, we note however that all regions of the country were represented, and all different academic levels in physiotherapy in Cameroon and both sexes were represented, which adds to the value of the study.

### Conclusion and implications

This study shows that Cameroonian physiotherapists’ general understanding of EBP is below average and describes a lack of knowledge, confidence and implementation of EBP in clinical practice and teaching. The main barriers to EBP implementation among physiotherapists in Cameroon were lack of time, workload and difficulty in accessing up-to-date information resources (databases). Our results could have a positive impact on the quality of training of physiotherapy students in Cameroon and on raising the level of scientific research in physiotherapy in order to promote the implementation of EBP for a better valorization of physiotherapy in Cameroon. To the extent that, the concept of EBP in physiotherapy is taught and widely used in many countries around the world, and we see that it is being developed in these countries, which are increasingly emphasising the need to develop research in physiotherapy. This is the case, for example, in France, England and many others.

### Suggestions

We encourage the Cameroonian Government to recruit more physiotherapists and assign them to all the country’s public hospitals which will help to reduce the workload of physiotherapists and therefore permit an adequate work environment.

We also suggest to The Cameroon Society of Physiotherapy (CASP), Association of Physiotherapists from Dschang University (APDU) and other associations working to promote the profession to organize continuous training seminars for physiotherapists on EBP, including the use of scientific databases and an understanding of EBP fields. Moreover we propose to The Ministry of Public Health and Ministry of Higher Education to introduce EBP modules as obligatory courses in the training program of physiotherapists in Cameroon, and to all and to all academic institutions involved in the training of physiotherapy students to do something to pronate EBP. Otherwise, we encourage physiotherapists who have adequate knowledge on EBP to continue upgrading their knowledge as science is rapidly evolving and most importantly to implement EBP in their daily practice. Finally, we encourage all practising physiotherapists to become increasingly involved in research and publication of scientific articles which will inform the national and international community on the advances in physiotherapy in Cameroon.

## Data Availability

The datasets used and/or analysed during the current study available from the corresponding author on reasonable request.

## Abbreviations

APDU: Association of Physiotherapists of Dschang University
CASP: Cameroon Society of Physiotherapy
DU: “Diplôme d’Université”
EBM: Evidence-Based Medicine
EBP: Evidence-Based Practice
EBP2: Evidence-BAsed Practice Profile
IBM: International Business Machines
PEDro: Physiotherapy Evidence Database
SD: Standard Deviation
SPSS: Statistical Package for Social Sciences
UAE: United Arabe Emirates
WP: World Physiotherapy
